# Influenza B associated paediatric acute respiratory infection hospitalization in central vietnam

**DOI:** 10.1111/irv.12626

**Published:** 2019-02-28

**Authors:** Keisuke Yoshihara, Minh Nhat Le, Michiko Toizumi, Hien Anh Nguyen, Hien Minh Vo, Takato Odagiri, Seiichiro Fujisaki, Koya Ariyoshi, Hiroyuki Moriuchi, Masahiro Hashizume, Duc Anh Dang, Lay‐Myint Yoshida

**Affiliations:** ^1^ Department of Paediatric Infectious Diseases Institute of Tropical Medicine Nagasaki University Nagasaki Japan; ^2^ National Institute of Hygiene and Epidemiology Hanoi Vietnam; ^3^ Khanh Hoa General Hospital Nha Trang Vietnam; ^4^ Influenza Virus Research Center National Institute of Infectious Diseases Tokyo Japan; ^5^ Department of Clinical Medicine Institute of Tropical Medicine Nagasaki University Nagasaki Japan; ^6^ Graduate School of Biomedical Sciences Nagasaki University Nagasaki Japan; ^7^ Department of Paediatrics Nagasaki University Hospital Nagasaki Japan

**Keywords:** acute respiratory infections, influenza B, molecular epidemiology, paediatric infectious diseases, Vietnam

## Abstract

**Background:**

Influenza B is one of the major etiologies for acute respiratory infections (ARI) among children worldwide; however, its clinical‐epidemiological information is limited. We aimed to investigate the hospitalization incidence and clinical‐epidemiological characteristics of influenza B‐associated paediatric ARIs in central Vietnam.

**Methods:**

We collected clinical‐epidemiological information and nasopharyngeal swabs from ARI children hospitalized at Khanh Hoa General Hospital, Nha Trang, Vietnam from February 2007 through June 2013. Nasopharyngeal samples were screened for 13 respiratory viruses using Multiplex‐PCRs. Influenza B‐confirmed cases were genotyped by Haemagglutinin gene sequencing. We analyzed the clinical‐epidemiological characteristics of influenza B Lineages (Victoria/Yamagata) and WHO Groups.

**Results:**

In the pre‐A/H1N1pdm09 period, influenza B‐associated ARI hospitalization incidence among children under five was low, ranging between 14.7 and 80.7 per 100 000 population. The incidence increased to between 51.4 and 330 in the post‐A/H1N1pdm09. Influenza B ARI cases were slightly older with milder symptoms. Both Victoria and Yamagata lineages were detected before the A/H1N1pdm09 outbreak; however, Victoria lineage became predominant in 2010‐2013 (84% Victoria vs 16% Yamagata). Victoria and Yamagata lineages did not differ in demographic and clinical characteristics. In Victoria lineage, Group1 ARI cases were clinically more severe compared to Group5, presenting a greater proportion of wheeze, tachypnea, and lower respiratory tract infection.

**Conclusions:**

The current results highlight the increased incidence of influenza B‐related ARI hospitalization among children in central Vietnam in the post‐A/H1N1pdm09 era. Furthermore, the difference in clinical severity between Victoria lineage Group1 and 5 implies the importance of influenza B genetic variation on clinical presentation.

## 1 INTRODUCTION

Influenza viruses belong to the family *Orthomyxoviridae,* which possess a segmented negative‐stranded RNA genome.^1^, ^2^ Among three influenza types, namely, type‐ A, B and C, influenza A and B often cause seasonal acute respiratory infections (ARI) epidemics and impose a high socioeconomic burden, particularly among children and the elderly population.^3^, ^4^ In temperate climate regions, peaks of influenza‐associated ARIs may appear in early autumn through winter,^5^ whereas year‐round circulation with no apparent seasonal trend may be seen in tropical climate regions.^6^, ^7^, ^8^, ^9^

Influenza B differs from type‐ A in terms of genetic composition and its ability to infect only humans and seals.^10^, ^11^ Two surface glycoproteins, Haemagglutinin (HA) and Neuraminidase (NA), play pivotal roles during pathogenesis.^12^ Unlike influenza A, influenza B does not possess multiple subtypes. Instead, influenza B obtains its genetic variation mainly through genetic drift, including nucleotide substitutions, insertions, and deletions, which explains the slower molecular evolutionary rate and smaller capacity to cause seasonal ARI outbreaks compared to influenza A.^13^, ^14^ Genetic reassortment of influenza B genetic components was previously documented^15^; however, its frequency is not as high as that of influenza A due to its limited animal reservoir.

Despite the clinical importance of influenza B among ARI cases, majority of previous literature focused on influenza A. Therefore, clinical and molecular epidemiological information on influenza B is relatively limited worldwide.

The first influenza B strain B/Lee/40 was isolated in 1940.^16^ In the 1970s, influenza B diverged into two genetically and antigenically distinct lineages, Victoria‐like (B/Victoria/2/87) and Yamagata‐like (B/Yamagata/16/88) lineages.^17^, ^18^ Since then, the two lineages have been co‐circulating in many regions of the world.^19^ Previous studies from Cambodia, China, India, and Taiwan have reported the increase of influenza B‐associated ARI cases in the following seasons after the emergence of the pandemic A/H1N1pdm09 strain.^4^, ^17^, ^18^, ^20^, ^21^ Furthermore, some studies presented the circulation of Victoria lineage as a dominant type in the post‐A/H1N1pdm09 period,^17^, ^18^, ^20^, ^21^, ^22^, ^23^ whereas others showed co‐circulation of both lineages.^3^, ^4^ Regardless of a few epidemiological studies on influenza B lineage‐specific clinical presentation,^21^, ^23^ the clinical aspect of two genetically distinct lineages has not been clearly understood up to date.

In this study, we investigated the incidence and clinical‐epidemiological characteristics of paediatric hospitalized influenza B ARI cases in Vietnam.

## 2 METHODS

### 2.1 Study site, period, and case enrollment

Population‐based prospective paediatric ARI surveillance in Khanh Hoa province, Nha Trang, Vietnam was established in February 2007.^24^ All children from 16 communes admitted to the paediatric ward of Khanh Hoa General Hospital (KHGH), which is the only hospital in the region, due to ARI symptoms (cough and/or difficulty breathing) from February 2007 to June 2013 were enrolled in the current study. Nha Trang city in south central region of Vietnam has a hot and dry season or tropical climate with a short rainy or wet season from September to December. The detailed characterization of the target population was described in the previous study.^24^ Written informed consent from the parents or guardians of all the enrolled ARI children were obtained. Nasopharyngeal (NP) swab, clinical‐epidemiological information, chest radiograph, and laboratory test data were collected from each enrollee. Currently, vaccination against influenza type A and B is not included in the nationwide immunization program in Vietnam. Its availability in our study site is limited and not commonly used among children.

### 2.2 Clinical information categorization

Lower respiratory tract infection (LRTI) was defined based on the modified World Health Organization (WHO) Integrated Management of Childhood Illnesses (IMCI) algorithms.^25^ The presence of tachypnea (respiratory rate >60/min for children ≦1 month, >50/min for 2‐11 months and >40/min for 12‐59 months of age) was categorized as mild LRTIs. Furthermore, children with a general danger sign (the situation in which children were either unable to drink, under convulsion, or lethargy), chest‐wall indrawing, or stridor were categorized as severe LRTIs. Radiologically‐confirmed pneumonia was defined as the presence of either substantial alveolar consolidation or pleural effusion in chest X‐ray result following the standardized interpretation method established by WHO Vaccine Trial Investigators Group.^26^ ARI cases with abnormal shadow but neither substantial alveolar consolidation nor pleural effusion were categorized into chest X‐ray abnormality or other lower respiratory infections.^26^

### 2.3 Viral screening and influenza B sequencing

Viral nucleic acids were extracted from ARI patient's NP swabs using QIA Viral RNA Minikit (QIAGEN Inc., Valencia, CA, USA) following the company's instructions. Four multiplex Reverse Transcriptase (RT)‐PCR assays were performed for screening 13 respiratory viruses, including RSV, influenza A and B, Human Metapneumovirus, Parainfluenza virus 1‐4, Human Coronavirus (229E and OC43), Adenovirus, and Bocavirus as previously described.^24^ For the current study, the presence of bacterial pathogens in the clinical samples was not tested.

Influenza B‐confirmed ARI samples were genotyped by Haemagglutinin (*HA*) gene sequencing using BigDye Terminator ver.3.1 cycle sequencing kit (Applied Biosystem, Foster City, CA, USA) with 3730 DNA Analyzer (Applied Biosystem, Foster City, CA, USA). Influenza B reference sequences including WHO recommended vaccine strains for northern hemisphere from 2007 to 2014 (https://www.who.int/influenza/vaccines/virus/recommendations/en/) were obtained from GenBank (http://www.ncbi.nlm.nih.gov/genbank/) and EpiFlu database within Global Initiative on Sharing All Influenza Data (GISAID)(http://platform.gisaid.org) and included in phylogenetic analysis as previously described.^17^, ^23^, ^27^ These reference strains were used in order to further classify lineage into groups based on Worldwide Influenza Centre (WIC) (https://www.crick.ac.uk/partnerships/worldwide-influenza-centre). Multiple nucleotide sequences of *HA* gene were aligned and edited with ClustalW algorism implemented within BioEdit ver.7.2.0. KAKUSAN4 (http://www.fifthdimension.jp/products/kakusan/) was used for the selection of best‐fit nucleotide substitution model.^28^ Phylogenetic analyses were performed with Maximum‐Likelihood (ML) method under HKY‐85 substitution model (gamma‐distribution) with bootstrap value of 1000 replicates using MEGA ver.6.0.6.^29^

### 2.4 Statistical analysis

Either two‐tailed Pearson's chi‐squared or Fisher's exact test was performed to compare the proportions between independent groups. For continuous variables, either Mann–Whitney *U* test or Kruskal–Wallis test were utilized to compare the median values between independent groups. In the multivariate analysis, Generalized Linear Model with log‐binomial regression was applied to estimate adjusted Relative Risk (Adj RR) and 95% Confidence Interval (CI). To control demographic confounding variables in the multivariate regression analysis, both forward‐selection step and biologically plausible approaches were taken into account. For the estimation of potential impact of A/H1N1pdm09 emergence on influenza B‐associated ARI hospitalization incidence among Vietnamese children, we utilized the Generalized Linear Model (GLM) with Poisson distribution.^30^ The following equation was used as a standard regression model:

$$Y_{t}∼\text{Poisson}\left(\mu_{t}\right)$$

$$\text{log}\left(\mu_{t}\right)=\beta_{0}+\beta_{1}T+\beta_{2}x_{t}+f\left(t\right)$$

1. *Y*_t_: the number of influenza B‐associated ARI hospitalization cases at time (*t*)
2. *T*: the time (in month‐interval) elapsed since the beginning of paediatric ARI surveillance in central Vietnam (February, 2007)
3. *X*_*t*_: the variable for “pre‐A/H1N1pdm09” (February 2007‐March 2010)(coded as 0) and “Post‐A/H1N1pdm09” (April 2010‐June 2013)(coded as 1)

where the function of *f* (*t*) adjusts for short‐term seasonal trend using the Fourier term (pairs of sine‐cosine functions) with four harmonic terms (2 sine‐cosine pairs). The values of Akaike Information Criterion (AIC) in the models with different numbers of harmonic terms [up to 12 (6 sine‐cosine pairs)] were compared in order to determine the best‐fit harmonic terms in the final model.^31^ The “Post‐A/H1N1pdm09” was defined as (April 2010‐June 2013), based on the AIC comparison in 12 different models (1 month interval), in which “Post‐A/H1N1pdm09” was defined at different time points, starting from January 2010 to December 2010. All the statistical analyses were performed using STATA ver.14.1 (StataCorp LP, College Station, TX, USA). *P*‐values less than 0.05 were considered to be statistically significant.

### 2.5 Ethics

This study was approved by the institutional ethical review boards of the National Institute of Hygiene and Epidemiology (NIHE), Hanoi, Vietnam (Approved Number: IRB‐VN01057), and the Institute of Tropical Medicine, Nagasaki University, Japan (Approved Number: 09031837). The study was conducted in accordance with the approved guidelines.

## 3 RESULTS

### 3.1 Yearly incidences of paediatric ARI hospitalizations, LRTIs, and influenza (A/B) ARIs

During the investigation period, February 2007 through June 2013, a total of 4429 paediatric ARI hospitalization cases were enrolled (Table 1), among which 496 were influenza A (single infection), and 129 were influenza B (single infection). Four cases were infected with both influenza A and B. Yearly incidences of paediatric ARI hospitalizations, LRTIs, and overall influenza‐associated ARI hospitalizations (both influenza A and B inclusive) were summarized in Table 1. The age‐stratified prevalence of influenza A and B among paediatric ARI hospitalizations were summarized in Table S1.

**Table 1:** Yearly incidences of paediatric ARI hospitalizations, LRTIs, and influenza (A/B) ARIs

	Overall	2007 (Feb‐Dec)	2008 (Jan‐Dec)	2009 (Jan‐Dec)	2010 (Jan‐Dec)	2011 (Jan‐Dec)	2012 (Jan‐Dec)	2013 (Jan‐Jun)
	Incidence per 100 000 population (95% Confidence interval)							
Paediatric ARI hospitalizations (n = 4429)	1507 (1401‐1619)	1687 (1574‐1806)	1281 (1182‐1385)	1548 (1440‐1662)	1146 (1053‐1245)	1065 (975‐1161)	1672 (1560‐1791)	2153 (2025‐2286)
Lower respiratory tract infections (n = 1075)	358 (308‐413)	481 (421‐547)	216 (176‐261)	309 (261‐362)	232 (191‐279)	133 (102‐169)	562 (497‐632)	572 (507‐643)
Influenza (A & B inclusive) ARI cases (n = 629)	198 (161‐242)	267 (223‐318)	182 (146‐225)	211 (172‐257)	143 (111‐181)	122 (93.1‐158)	207 (169‐252)	255 (212‐304)
Influenza types (A/B)	Incidence per 100 000 population (95% Confidence interval)							
Influenza type A (single) (n = 496)	158 (126‐197)	263 (219‐313)	160 (126‐199)	189 (152‐232)	74.6 (52.3‐103)	106 (78.7‐139)	93.2 (68.0‐125)	224 (184‐270)
Less than 2 years old (n = 281)	808 (596‐1074)	1491 (1188‐1847)	636 (444‐884)	818 (597‐1093)	418 (265‐627)	473 (309‐692)	418 (265‐627)	1400 (1106‐1747)
Less than 5 years old (n = 451)	511 (402‐643)	895 (744‐1068)	477 (368‐607)	572 (453‐714)	242 (167‐340)	345 (254‐458)	308 (222‐416)	741 (604‐900)
Older than 5 years old (n = 45)	19.4 (8.2‐40.0)	14.4 (4.7‐33.7)	34.7 (17.9‐60.5)	37.5 (20.0‐64.2)	8.7 (1.8‐25.3)	11.6 (3.2‐29.6)	8.7 (1.8‐25.3)	20.2 (8.1‐41.6)
Influenza type B (single) (n = 129)	39.1 (25.1‐59.7)	4.1 (0.5‐15.0)	22.8 (11.4‐40.8)	22.8 (11.4‐40.8)	66.3 (45.4‐93.6)	16.6 (7.2‐32.7)	110 (82.3‐144)	31.1 (17.4‐51.3)
Less than 2 years old (n = 66)	182 (98.4‐325)	18.2 (0.5‐101)	164 (74.9‐310)	90.9 (29.5‐212)	255 (139‐427)	36.4 (4.4‐131)	527 (353‐756)	182 (87.2‐334)
Less than 5 years old (n = 109)	117 (71.9‐186)	14.7 (1.8‐53.0)	73.4 (35.2‐135)	80.7 (40.3‐144)	183 (119‐271)	51.4 (20.7‐106)	330 (241‐442)	88.0 (45.5‐154)
Older than 5 years old (n = 20)	0.4 (0‐12.2)	0 (0‐10.7)	0 (0‐10.7)	0 (0‐10.7)	2.9 (0.1‐16.1)	0 (0‐10.7)	0 (0‐10.7)	0 (0‐10.7)
Influenza A and B (co‐detection) (n = 4)	0.9 (0.1‐9.2)	0 (0‐7.6)	0 (0‐7.6)	0 (0‐7.6)	2.1 (0.1‐11.5)	0 (0‐7.6)	4.1 (0.5‐15.0)	0 (0‐7.6)

The incidence of influenza A and B‐associated paediatric ARI hospitalization were highest among children younger than 2 years (Table 1). During the first three years of the study period (2007‐2009), the incidence of influenza B ARI hospitalization among children younger than two were relatively low, ranging from 18.2 to 164 per 100 000 population compared to influenza A, which was 636 to 1491 per 100 000. However, the incidence of influenza B ARI hospitalization among children younger than two increased noticeably in the post‐A/H1N1pdm09 period, particularly in 2010 [255 (95% CI: 139‐427)] and 2012 [527 (95% CI: 353‐756)]. The results of Generalized Linear Model (GLM) confirmed that there was statistical evidence of an increase in influenza B‐associated paediatric ARI hospitalization in “Post‐A/H1N1pdm09” (April 2010‐June 2013) when compared to “Pre‐A/H1N1pdm09” (February 2007‐March 2010) [Incidence Relative Risk (IRR) 3.12, 95% CI: 1.01‐9.61, *P *=* *0.047] _ENREF_17. On the other hand, there was no statistical evidence of an increase in influenza A‐related ARI hospitalization incidence in post‐A/H1N1pdm09 compared to Pre‐A/H1N1pdm09 period [IRR 0.69, 95% CI: 0.35‐1.38, *P *=* *0.293].

### 3.2 Demographic and clinical characteristics comparison between influenza B and non‐influenza B ARIs

With regard to the demographic and clinical characterization of overall paediatric ARI hospitalization cases enrolled in the current study (n = 4,429), 2,602 were male (58.8%), and the median age (in months) was 16.6 (IQR: 8.6‐27.3). Other demographic information, including socioeconomic status, medical history, as well as detailed clinical information, was summarized in Table 2.

**Table 2:** Demographic and clinical characteristics comparison between influenza B and non‐influenza B ARI cases

	Total number of paediatric ARI hospitalizations in KHGH during February 2007‐June 2013 (n = 4429)			
	Overall paediatric ARI cases (n = 4429)	Inf B positive ARI group (n = 133)	Non‐inf B ARI group (n = 4296)	*P*‐value^d^
	Total number (%)/Median (IQR)^a^			
Demographic information				
Male sex (%)	2602 (58.8%)	67 (50.4%)	2535 (59.0%)	**0.046** *
Median age (in month)	16.6 (IQR: 8.6‐27.3)	22.9 (IQR: 12.9‐48.7)	16.5 (IQR: 8.5‐26.9)	**<0.001** ***
Age group (%)				
0‐12 month	1721 (38.9%)	34 (25.6%)	1687 (39.3%)	**<0.001** ***
13‐24 month	1435 (32.4%)	39 (29.3%)	1396 (32.5%)	
25‐36 month	609 (13.8%)	12 (9.0%)	597 (13.9%)	
37‐48 month	246 (5.6%)	16 (12.0%)	230 (5.4%)	
49‐60 month	132 (3.0%)	10 (7.5%)	122 (2.8%)	
>60 month	286 (6.5%)	22 (16.5%)	264 (6.2%)	
Socioeconomic status				
Daycare attendance (%)	1828 (41.3%)	61 (45.9%)	1767 (41.1%)	0.285
Family smoking (%)	2273 (51.3%)	87 (65.4%)	2186 (50.9%)	**0.003** **
History				
Antibiotic used prior to hospitalization (%)	1699 (38.4%)	53 (39.9%)	1646 (38.3%)	0.885
Underlying medical condition (%)	1681 (38.0%)	51 (38.4%)	1630 (37.9%)	0.925
Clinical information				
Vital sign(s)				
Median respiratory rate (per min)	33.0 (IQR: 30.0‐40.0)	32.0 (IQR: 29.0‐37.0)	33.0 (IQR: 30.0‐40.0)	**0.012** *
Median body temperature (°C)	38.0 (IQR: 37.2‐38.5)	38.0 (IQR: 37.5‐38.8)	38.0 (IQR: 37.2‐38.5)	0.571
SpO2 (≦90%)	220 (5.0%)	5 (3.8%)	215 (5.0%)	0.685
Respiratory symptom and sign(s)				
Wheeze (%)	2300 (51.9%)	53 (39.9%)	2247 (52.3%)	**0.005** **
Tachypnea (%)	889 (20.1%)	28 (21.1%)	861 (20.0%)	0.774
Breathing difficulty (%)	548 (12.4%)	8 (6.0%)	540 (12.6%)	**0.022** *
Crackle (%)	470 (10.6%)	20 (15.0%)	450 (10.5%)	0.092
LRTI and chest X‐ray result				
LRTI^b^(%)	1041 (23.5%)	27 (20.3%)	1014 (23.6%)	0.376
Mild LRTI (%)	676 (15.3%)	19 (14.3%)	657 (15.3%)	0.662
Severe LRTI^c^(%)	365 (8.2%)	8 (6.0%)	357 (8.3%)	0.421
Abnormal chest X‐ray (%)	1267 (28.6%)	20 (15.0%)	1247 (29.0%)	**<0.001** ***
Radiologically‐confirmed pneumonia (%)	790 (17.8%)	15 (11.3%)	775 (18.0%)	0.052
Treatment and outcome(s)				
Median onset to hospitalization (in day)	2.0 (IQR: 1.0‐3.0)	2.0 (IQR: 1.0‐3.0)	2.0 (IQR: 1.0‐3.0)	0.897
Median hospitalization duration (in day)	4.0 (IQR: 3.0‐6.0)	4.0 (IQR: 2.0‐6.0)	4.0 (IQR: 3.0‐6.0)	**0.037** *
Antibiotic used (%)	4364 (98.5%)	132 (99.3%)	4232 (98.5%)	1.000
Steroid used (%)	2116 (47.8%)	57 (42.9%)	2059 (47.9%)	0.248
Blood WBC count (10^3^ cells/uL)	11.4 (IQR: 8.6‐15.2)	9.2 (IQR: 7.0‐12.9)	11.5 (IQR: 8.6‐15.2)	**<0.001** ***

a IQR is an abbreviation for Interquartile Range (1st to 3rd).

b LRTI is an abbreviation for “lower respiratory tract infection” and based on the WHO definition of clinical pneumonia24.

c Severe LRTI was defined as the presence of a danger sign, stridor, or chest‐wall indrawing.

d Proportions and median values were compared between influenza B (n = 133) and non‐influenza B (n = 4296) ARI groups. All the statistically significant *P*‐values are indicated in bold font. As the index for statistically significant values: * are used for *P*‐values <0.05, ** for *P*‐values <0.01, and *** for *P*‐values ≦0.001.

The demographic and clinical characteristics between influenza B (n = 133) and non‐influenza B ARI groups (n = 4296) were compared (Table 2). The male proportion was higher in the non‐influenza B ARI group (50.4%, influenza B vs 59.0%, non‐influenza B, *P *=* *0.046). Regarding the median age (in month), the influenza B ARI group was older (22.9, influenza B vs 16.5, non‐influenza B, *P *<* *0.001), and the greater proportion of ARI children were 3 years or older in the influenza B ARI group. Furthermore, family smoking was more frequent in the influenza B ARI group (*P *=* *0.003).

With respect to the clinical information, the respiratory rate (per min) was faster in the non‐influenza B ARI group (32.0, influenza B vs 33.0, non‐influenza B, *P *=* *0.012) (Table 2). Furthermore, ARI children with wheeze (*P *=* *0.005) and breathing difficulty (*P *=* *0.022) were more common in the non‐influenza B ARI group. The proportion of paediatric ARI hospitalizations with chest X‐ray abnormal findings was also greater in the non‐influenza B ARI group (15.0%, influenza B vs 29.0%, non‐influenza B, *P *<* *0.001). The result of multivariate analyses presented that non‐influenza B ARI cases were associated with the increased risks of respiratory clinical presentations after adjusting for sex, age, and family smoking status. For instance, the risk of wheeze was 1.51 (95% CI: 1.07‐2.13) times, breathing difficulty was 2.21 (95% CI: 1.09‐4.48) times, and chest X‐ray abnormality was 2.56 (95% CI: 1.54‐4.25) times greater in non‐influenza B ARI cases. The median WBC titre was higher in the non‐influenza B ARI group (9.2 × 10^3^ cells/uL, influenza B vs 11.5 × 10^3^ cells/uL, non‐influenza B, *P *<* *0.001).

Furthermore, in the comparison between influenza A and B, influenza B‐associated ARI hospitalizations were slightly older with different age distribution pattern (*P *=* *0.002) (Table S2). Parental smoking was more common in influenza B ARI group (*P *=* *0.011). Regarding the clinical information, influenza A group was associated with higher body temperature (*P *=* *0.003) as well as frequent presence of chest X‐ray abnormality (*P *<* *0.001).

### 3.3 Yearly prevalence and seasonality of influenza B Victoria and Yamagata lineages

Among 133 influenza B‐associated paediatric ARI hospitalization cases detected from February 2007‐June 2013, a total of 106 influenza B samples were analyzed for Haemagglutinin (HA) glycoprotein nucleotide sequences. Of 106 influenza B *HA* gene sequenced ARI samples, 91 (85.9%) were successfully sequenced and utilized for phylogenetic analysis.

**Figure 1:**
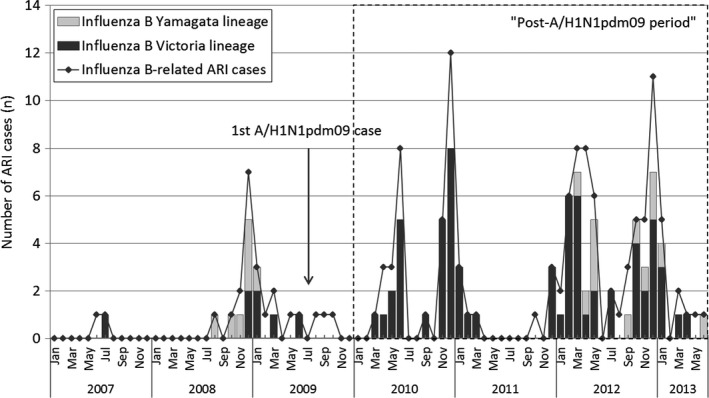
Seasonal circulation pattern of influenza B lineages (Victoria/Yamagata) from February 2007 to June 2013. A cumulative number of influenza B paediatric ARI hospitalizations in each month was presented as the BLACK solid line. Victoria lineage ARI case in each month was shown in the BLACK box and Yamagata lineage ARI cases in the GREY box. The first A/H1N1pdm09‐related ARI hospitalization case detected in the current study site was indicated with the down arrow. The period encompassing January 2010‐June 2013 was defined as “Post‐A/H1N1pdm09 period” and shown as a dotted‐box

Regarding the influenza B lineage‐specific seasonal trend during the current investigation period, no distinct trend was observed in the pre‐A/H1N1pdm09 period (Figure 1). However, starting from post‐A/H1N1pdm09, two peaks of influenza B‐associated ARIs appeared within a year: from February through June and from October through December (Figure 1), which was mainly contributed by Victoria lineage‐related ARI hospitalizations, particularly in 2010 (n = 28) and 2012 (n = 37) (Table S3). On the contrary, the overall number of Yamagata lineage‐related ARI hospitalizations was limited throughout the investigation period in both pre‐ and post‐A/H1N1pdm09 periods.

### 3.4 Demographic and clinical characteristics comparison between Victoria and Yamagata lineages

In the demographic characteristics comparison between influenza B Victoria (n = 72) and Yamagata (n = 19) lineages, the median age (in month) was older among Victoria lineage ARI cases (25.8, Victoria lineage vs 19.7, Yamagata lineage, *P *=* *0.056) with a slightly higher proportion of ARIs in the 2 years and older age group in Victoria lineage (*P *=* *0.126) (Table 3). Furthermore, kindergarten attendance was more frequent in the Victoria lineage ARI group (55.6%, Victoria lineage vs 21.1%, Yamagata lineage, *P *=* *0.010). ARI hospitalizations, with the presence of underlying medical conditions and co‐infection with other respiratory viruses, were common in both lineages.

**Table 3:** Demographic and clinical characteristics comparison between influenza B Victoria and Yamagata lineages

	Influenza B positive paediatric ARI cases during February 2007‐June 2013 (n = 133)		
	Influenza B Victoria lineage (n = 72)	Influenza B Yamagata lineage (n = 19)	
	Total number (%)/Median (IQR)^a^		*P*‐value
Demographic information			
Male sex (%)	41 (56.9%)	9 (47.4%)	0.605
Median age (in month)	25.8 (IQR: 13.7‐51.5)	19.7 (IQR: 7.1‐25.2)	0.056
Age group (%)			
0‐12 month	14 (19.4%)	7 (36.8%)	0.126
13‐24 month	21 (29.2%)	7 (36.8%)	
25‐36 month	5 (6.9%)	2 (10.5%)	
37‐48 month	12 (16.7%)	0	
49‐60 month	6 (8.3%)	2 (10.5%)	
>60 month	14 (19.4%)	1 (5.3%)	
Socioeconomic status			
Daycare attendance (%)	40 (55.6%)	4 (21.1%)	**0.010** *
Family smoking (%)	48 (66.7%)	12 (63.2%)	0.573
History			
Antibiotic used prior to hospitalization (%)	31 (43.1%)	7 (36.8%)	0.412
Underlying medical condition (%)	26 (36.1%)	5 (26.3%)	0.588
Respiratory virus co‐infection (%)	15 (20.8%)	3 (15.8%)	0.755
Clinical information			
Vital sign(s)			
Median respiratory rate (per min)	30.0 (IQR: 28.0‐35.0)	34.0 (IQR: 30.0‐40.0)	0.153
Median body temperature (°C)	37.6 (IQR: 37.0‐38.6)	38.5 (IQR: 37.6‐39.0)	**0.017** *
SpO2 (≦90%)	1 (1.4%)	3 (15.8%)	**0.028** *
Respiratory symptom and sign(s)			
Wheeze (%)	26 (36.1%)	11 (57.9%)	0.116
Tachypnea (%)	13 (18.1%)	5 (26.3%)	0.518
Breathing difficulty (%)	5 (6.9%)	1 (5.3%)	1.000
Crackle (%)	9 (12.5%)	3 (15.8%)	0.709
LRTI and chest X‐ray result			
LRTI^b^(%)	12 (16.7%)	3 (15.8%)	1.000
Mild LRTI (%)	9 (12.5%)	2 (10.5%)	1.000
Severe LRTI^c^(%)	3 (4.2%)	1 (5.3%)	1.000
Abnormal chest X‐ray (%)	10 (13.9%)	5 (26.3%)	0.305
Radiologically‐confirmed pneumonia (%)	8 (11.1%)	3 (15.8%)	0.701
Treatment and outcome(s)			
Median onset to hospitalization (in day)	2.0 (IQR: 1.0‐4.0)	1.0 (IQR: 1.0‐3.0)	0.176
Median hospitalization duration (in day)	4.0 (IQR: 2.0‐6.0)	4.0 (IQR: 2.0‐5.0)	0.507
Antibiotic used (%)	72 (100%)	18 (94.7%)	0.209
Steroid used (%)	31 (43.1%)	7 (36.8%)	0.795
Blood WBC count (10^3^ cells/uL)	9.4 (IQR: 6.9‐13.2)	8.8 (IQR: 6.7‐12.8)	0.965

a IQR is an abbreviation for Interquartile Range (1st to 3rd).

b LRTI is an abbreviation for “lower respiratory tract infection” and based on the WHO definition of clinical pneumonia.^24^

c Severe LRTI was defined as the presence of a danger sign, stridor, or chest‐wall indrawing.

All the statistically significant *P*‐values are indicated in bold font. As the index for statistically significant values: * are used for *P‐*values <0.05, ** for *P*‐values <0.01, and *** for *P*‐values ≦0.001.

Regarding the clinical manifestation comparison between influenza B lineages, SpO2 (≦90%) was more commonly seen in the Yamagata lineage ARI group (15.8%, Yamagata lineage vs 1.4%, Victoria lineage, *P *=* *0.028) (Table 3). Furthermore, the median body temperature (°C) was higher in the Yamagata lineage (38.5, Yamagata lineage vs 37.6, Victoria lineage, *P *=* *0.017). Other respiratory‐related clinical information, such as the presence of wheeze, tachypnea, and chest X‐ray abnormal findings were also relatively more common in Yamagata lineage ARI group with no statistically significant differences.

### 3.5 Seasonal prevalence of WHO Groups in Victoria and Yamagata lineages

**Figure 2:**
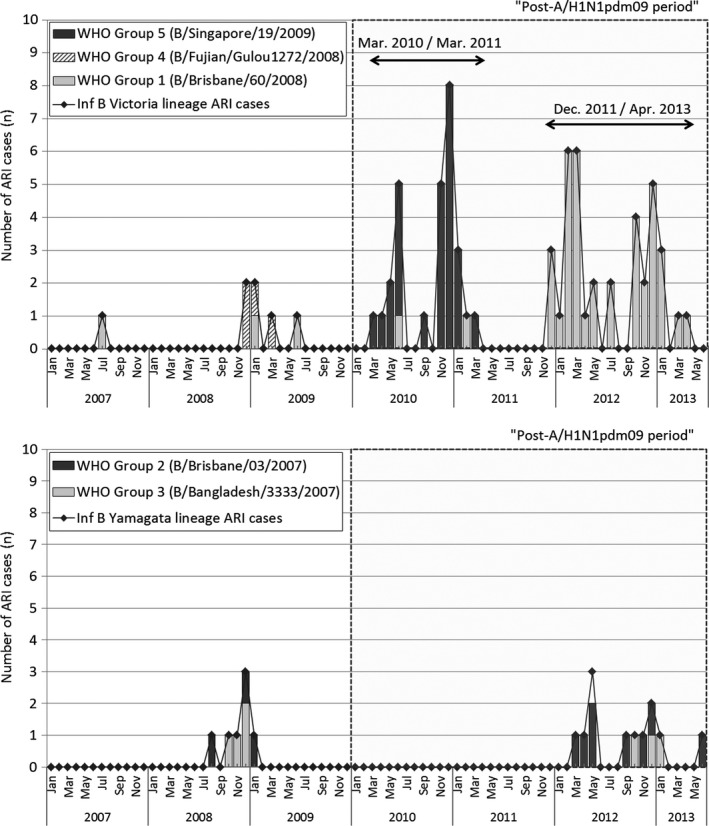
Seasonal circulation pattern of WHO Groups in Victoria lineage (top) and Yamagata lineage (bottom) from February 2007 to June 2013. A cumulative number of influenza B lineage‐specific paediatric ARI hospitalizations in each month was shown as the BLACK solid line. WHO Groups classification for each influenza B lineage is based on Worldwide Influenza Centre (WIC). WHO Group‐specific ARI hospitalizations were presented as the boxes with different colors: Victoria lineage (GREY for Group 1, GREY‐stripe for Group 4 and BLACK for Group 5) and Yamagata lineage (BLACK for Group 2 and GREY for Group 3). The period encompassing January 2010‐June 2013 was defined as “Post‐A/H1N1pdm09 period” and shown as dotted‐box

The seasonal prevalence of WHO Groups in both influenza B lineages, circulating during the present study period, was investigated. With respect to the Victoria lineage, a remarkable shift in the dominantly circulating WHO Group was detected in post‐A/H1N1pdm09 period: Group 5 (B/Singapore/19/2009) was the dominant type during Mar 2010‐Mar 2011, which was rapidly replaced by Group 1 (B/Brisbane/60/2008) in Dec 2011‐Apr 2013 (Figure 2).

Regarding the seasonal prevalence of Yamagata lineage WHO Group, Group 2 and 3 were detected with no particular circulation trend or shift in dominant Group over the course of investigation period. A detailed seasonal prevalence of WHO Groups in both influenza B lineages was summarized in Table S3.

### 3.6 Demographic and clinical characteristics comparison among WHO Groups of Victoria and Yamagata lineages

Firstly, demographic information among WHO Groups (1, 4, and 5) of Victoria lineage was compared (Table 4). There were no significant differences in sex distribution and median age among the three groups. Daycare attendance was more common in Group 5 compared to Group 1 (73.1%, Group 5 vs 45.2%, Group 1, *P *=* *0.043). Furthermore, the proportion of hospitalized ARI children with antibiotic usage prior to the hospital admission (*P *<* *0.001) and the presence of underlying medical conditions (*P *=* *0.002) was greater in Group 1 compared to Groups 4 and 5. In addition, co‐infections with other respiratory viruses were common in both Groups 1 and 5 (16.7%, Group 1 vs 30.8%, Group 5, *P *=* *0.231).

**Table 4:** Demographic and clinical characteristics comparison among WHO Groups (1, 4, and 5) in Victoria lineage

	Influenza B Victoria lineage paediatric ARI cases during February 2007‐June 2013 (n = 72)		
	WHO Group 1 (n = 42)	WHO Group 5 (n = 26)	
	Total number (%)/Median (IQR)^a^		*P*‐value
Demographic information			
Male sex (%)	23 (54.8%)	16 (61.5%)	0.622
Median age (in month)	28.1 (IQR: 13.2‐51.1)	25.0 (IQR: 17.5‐55.6)	0.601
Age group (%)			
0‐12 month	10 (23.8%)	2 (7.7%)	0.223
13‐24 month	10 (23.8%)	11 (42.3%)	
25‐36 month	2 (4.8%)	3 (11.5%)	
37‐48 month	9 (21.4%)	3 (11.5%)	
49‐60 month	4 (9.5%)	1 (3.9%)	
>60 month	7 (16.7%)	6 (23.1%)	
Socioeconomic status			
Daycare attendance (%)	19 (45.2%)	19 (73.1%)	**0.043** *
Family smoking (%)	26 (61.9%)	18 (69.2%)	0.578
History			
Antibiotic used prior to hospitalization (%)	23 (54.8%)	8 (30.8%)	**0.004** **
Underlying medical condition (%)	22 (52.4%)	4 (15.4%)	**0.002** **
Respiratory virus co‐infection (%)	7 (16.7%)	8 (30.8%)	0.231
Clinical information			
Vital sign(s)			
Median respiratory rate (per min)	32.0 (IQR: 29.0‐36.0)	30.0 (IQR: 28.0‐35.0)	0.272
Median body temperature (°C)	37.6 (IQR: 37.0‐39.0)	37.5 (IQR: 37.0‐38.0)	0.148
SpO2 (≦90%)	1 (2.4%)	0	1.000
Respiratory symptom and sign(s)			
Wheeze (%)	22 (52.4%)	4 (15.4%)	**0.002** **
Tachypnea (%)	12 (28.6%)	1 (3.9%)	**0.012** *
Breathing difficulty (%)	5 (11.9%)	0	0.148
Crackle (%)	9 (21.4%)	0	**0.010** *
LRTI and chest X‐ray result			
LRTI^b^(%)	10 (23.8%)	1 (3.9%)	**0.041** *
Mild LRTI (%)	7 (16.7%)	1 (3.9%)	0.131
Severe LRTI^c^(%)	3 (7.1%)	0	0.258
Abnormal chest X‐ray (%)	7 (16.7%)	2 (7.7%)	0.298
Radiologically‐confirmed pneumonia (%)	6 (14.3%)	2 (7.7%)	0.461
Treatment and outcome(s)			
Median onset to hospitalization (in day)	2.0 (IQR: 1.0‐4.0)	1.5 (IQR: 0‐4.0)	0.146
Median hospitalization duration (in day)	4.0 (IQR: 2.0‐6.0)	3.5 (IQR: 2.0‐5.0)	0.171
Antibiotic used (%)	42 (100%)	26 (100%)	1.000
Steroid used (%)	16 (38.1%)	15 (57.7%)	0.115
Blood WBC count (10^3^ cells/uL)	9.0 (IQR: 6.0‐12.4)	9.7 (IQR: 7.7‐13.4)	0.357

a IQR is an abbreviation for Interquartile Range (1st to 3rd).

b LRTI is an abbreviation for “lower respiratory tract infection” and based on the WHO definition of clinical pneumonia.^24^

c Severe LRTI was defined as the presence of a danger sign, stridor, or chest‐wall indrawing.

*P*‐values are for proportions and median values comparison between WHO Groups 1 and 5 of Victoria lineage.

All the statistically significant *P*‐values are indicated in bold font. As the index for statistically significant values: * are used for *P*‐values <0.05, ** for *P*‐values <0.01, and *** for *P*‐values ≦0.001.

With respect to clinical characterization of WHO Groups in Victoria lineage, wheeze (*P *=* *0.002), tachypnea (*P *=* *0.026), and crackle (*P *=* *0.019) were more commonly observed in Group 1 than in the other two groups (Table 4). Furthermore, hospitalized ARI cases with Group 1 presented the greater proportion of LRTIs compared to Group 5 (23.8%, Group 1 vs 3.9%, Group 5, *P *=* *0.041). Although statistically not significant, ARI children infected with Group 1 presented relatively higher proportions of chest X‐ray abnormality and radiologically‐confirmed pneumonia in comparison to Groups 4 and 5.

For demographic and clinical information comparison in Yamagata lineage WHO Groups, we could only include eleven ARI cases of Group 2 and seven of Group 3 in the statistical analyses (Table S4). With regard to demographic characterization, no statistically significant differences were detected between Groups 2 and 3. Both Group 2‐ and Group 3‐associated ARI hospitalizations were commonly observed among children younger than 2 years. The results of a comparison in clinical manifestations did not reveal any significant differences.

## 4 DISCUSSION

This is the first study to describe the detailed clinical‐epidemiological information of influenza B among hospitalized paediatric ARI cases in Vietnam. During the study period, both influenza A and B ARI hospitalizations were frequently detected in children younger than 2 years. The first A/H1N1pdm09‐associated ARI hospitalization case was reported in July 2009.^32^ Noticeably, the incidence of influenza B ARI hospitalization in the post‐A/H1N1pdm09 period increased dramatically, particularly in 2010 and 2012. Furthermore, Victoria lineage was dominant in post‐A/H1N1pdm09, and the dominantly circulating WHO Group shifted each year. Importantly, Victoria lineage WHO Group 1‐associated ARIs were clinically more severe in comparison with Group 5, presenting greater proportions in wheeze, tachypnea, and LRTI. Therefore, our current results highlight both the increased clinical importance of influenza B among paediatric ARI hospitalizations in central Vietnam in the post‐A/H1N1pdm09 period and the difference in respiratory‐related clinical severity among WHO Groups of Victoria lineage, which could help in the future nationwide influenza vaccination program and for proper clinical management in hospital settings.

During the current investigation period, overall influenza (A/B)‐associated paediatric ARI hospitalization incidence ranged from 122 to 267 (per 100 000 population) (Table 1). Both influenza A and B ARI incidences were high among children under 5 years. In fact, the proportion of hospitalized ARI children over 5 years was greater in the influenza B ARI group compared to influenza A (8.8%, influenza A vs 15.5%, influenza B, *P *=* *0.002) (data not shown), which was consistent with the previous reports from China and Finland.^3^, ^33^, ^34^ In the current study site, influenza B‐related paediatric ARI hospitalization incidence was low in Pre‐A/H1N1pdm09 period. However, influenza B ARI incidence dramatically increased in post‐A/H1N1pdm09, which resembled the previous reports from Brazil, Cambodia, China, India, and Taiwan.^4^, ^17^, ^18^, ^20^, ^21^, ^35^ As it was previously described, the emergence of A/H1N1pdm09 strain might have affected the viral etiology and seasonal circulation pattern of childhood ARIs.^36^ We concluded that the A/H1N1pdm09 outbreak occurred around mid‐2009 in our study site and assumed that a large population in the community might have been infected with this new A/H1N1pdm09 strain. This may have led to the development of non‐specific antiviral immunity in the target community, which may have disrupted the circulation trend of other respiratory viruses, including influenza B. Interestingly, we also observed unusual split of RSV seasonal peak in 2010 as we previously described.^37^ Further studies would be necessary to understand the effect of A/H1N1pdm09 on respiratory virus circulation trend.

Among all the paediatric ARI hospitalization cases enrolled in the current study, 133 were influenza B positive (Table 2). The median age in influenza B ARI group was older than non‐influenza B ARIs, and influenza B group presented the higher proportion of family smoking, which is known to be one of the major risk factors for influenza morbidity and mortality.^38^, ^39^ Passive smoking from family member might have an effect on suppression of humoral‐ and cell‐mediated immunity, which makes children more susceptible to influenza infections. However, the mechanism is not fully understood. Passive smoking in general may put children at the risk of ARI hospitalization due to viral infection; however, there is not yet clear evidence regarding the absolute effect of passive smoking on ARI hospitalization in different viral pathogens. The slightly older age of infection by influenza B compared to non‐influenza B (22.9 months vs 16.5 months, *P *<* *0.001) may explain an increased susceptibility by passive smoking in older age group. In the future study, we need to follow up children with positive passive smoking status in order to understand the effect of passive smoking on virus‐related ARI hospitalization in each virus respectively. Regarding clinical manifestation, influenza B ARI group was less severe than non‐influenza B, which might be due to the fact that influenza B ARI cases were relatively older. Also, the higher blood WBC titre in non‐influenza B ARI group was most likely due to a certain proportion of bacteria‐associated ARIs counted in non‐influenza B ARI group. In the comparison between influenza A and B, there were no remarkable differences except for the higher prevalence of parental smoking in influenza B ARI group (Table S2). Further study would be necessary to understand the effect of secondary smoking on susceptibility against influenza infection. In the clinical information comparison, influenza A group presented higher body temperature and frequent presence of chest X‐ray abnormality. Yet, other clinical presentations were similar between two. Although a study mentioned that influenza B patients frequently presented gastrointestinal symptoms,^40^ the actual difference in clinical severity between influenza A and B has been controversial up to date.^41^, ^42^, ^43^ Further continuous epidemiological surveillance would be necessary to gain a deeper understanding in the clinical aspect of influenza infection.

With respect to influenza B lineage specific seasonal trend in our study site, Victoria lineage was dominant in post‐A/H1N1pdm09 period (Figure 1), which was similar to studies from India, Cambodia, and Taiwan.^4^, ^17^, ^18^, ^20^ A previous report described Victoria lineage might possess greater viral transmissibility based on the estimated reproductive number (R*e*) as well as the faster molecular evolutionary rate compared to Yamagata lineage.^44^ The difference in viral transmissibility capacity between genetically distinct lineages may explain the predominance by Victoria lineage in the current study site. Furthermore, the difference in viral virulence may have played a role in increase of Victoria lineage‐associated ARI hospitalizations. We also have to take the lineage‐specific sero‐epidemiological information into account to gain a better understanding of influenza B lineage circulation pattern. A study from Taiwan suggested switching of dominantly circulating lineage may occur every 2‐3 years.^18^ In the future study, we will continue monitoring the lineage‐specific circulation trend of influenza B strains in order to investigate the lineage‐specific viral transmissibility based on the viral genetic variation.

Regarding the demographic information of influenza B lineages, median age was slightly older in Victoria lineage (Table 3), which contradicted the reports from China and Slovenia that presented Victoria lineage ARIs were younger than Yamagata lineage.^21^, ^45^ The difference in age of infection has been controversial due to the differences in inclusion criteria among studies. Furthermore, clinical aspect of influenza B lineages is poorly understood. In the current study, the clinical data did not present a significant difference between lineages (Table 3), which was in line with previous reports from China, France, Serbia, and Slovenia.^41^, ^45^, ^46^, ^47^, ^48^ Although the body temperature was slightly higher in Yamagata lineage ARIs, it was probably due to the difference in age distribution. In our study, the hospitalization duration did not differ between lineages, which contradicted the previous study that presented longer time to symptom resolution in Victoria lineage.^47^ Moreover, another study by Chi et al reported that Yamagata lineage ARIs were associated with invasive LRTI.^49^ Therefore, there exists inconsistency regarding the information of influenza B lineage‐specific demographic and clinical characteristics.

Regarding the seasonal circulation trend of WHO Groups in each lineage, we did not find a distinct circulation trend in the pre‐A/H1N1pdm09 period (Figure 2). However, starting from the post‐A/H1N1pdm09 period, WHO Group‐specific circulation trend became apparent in Victoria lineage, in which Group 5 was dominant during Mar 2010‐Mar 2011, and then followed by Group 1 in Dec 2011‐Apr 2013. In *HA* glycoprotein coding region that we targeted for nucleotide sequencing in the current study, Group 1 and 5 possess distinct amino acid (AA) substitutions at two sites within major antigenic regions: 120‐loop (Lysine (K) in Group 1 and Asparagine (N) in Group 5) and 160‐loop (Lysine (K) in Group 1 and Asparagine (N) in Group 5)(B/Brisbane/60/2008 strain (Accession number: FJ766840) as the reference of AA position).^50^ These two AA substitutions were located proximity to the biding site to SA‐galactose linkage, yet not within actual binding site. Therefore, genetically distinct groups may have different receptor binding affinities, viral transmissibility, which may have been linked with the sudden increase by Group 5 in Mar 2010‐Mar 2011. Further studies would be necessary to understand the role of differences in AA composition within major antigenic region on the receptor binding capacity and viral transmissibility.

Our current result was consistent with the previous finding that a dominantly circulating Group in 1 year would be replaced by another Group in the following year.^22^ To the best of our knowledge, this was the first study describing the dramatic shift in Groups between years in Vietnam.

Most importantly, ARI cases infected with Victoria lineage Group 1 were more frequently associated with severe clinical manifestations such as wheeze, tachypnea, crackle and LRTI in comparison with Group 4 and 5. One possible explanation is the distinct amino acid composition within *HA* gene of Victoria lineage Groups may possess Group‐specific binding preference to sialic acid (SA)‐galactose linkage, α2,3 or α2,6. It was previously described that few amino acid substitutions in HA glycoprotein could affect receptor binding specificity.^51^ For example, Asp‐225‐Gly substitution of A/H1N1pdm09 strain resulted in increased binding affinity to α2,3‐SA linkage, which enhanced disease severity.^52^, ^53^, ^54^ It is important to investigate the clinical role of two amino acid substitutions within major antigenic regions between Victoria lineage Group 1 and 5. Therefore, the pathological and clinical importance of Victoria lineage Group‐specific amino acid composition within HA glycoprotein antigenic regions needs to be further investigated.

As one of the limitations in the clinical manifestation comparison of WHO groups, we were not capable of performing multivariate analysis adjusting for confounding variables due to the small sample size in each Group. Since the presence of an underlying medical condition was described to be one of the major risk factors for developing severe influenza B‐related ARI infection,^46^ our primary clinical finding of the association of Victoria lineage Group 1 with clinical severity, should be carefully interpreted as its severity could have been effected by the higher prevalence of an underlying medical condition in Group 1. Co‐infection with other respiratory viruses should be taken into account in future analysis as the frequency of co‐infection with other respiratory pathogens were relatively high in both WHO Group 1 and 5, rate of which was comparable to previous studies.^55^, ^56^ Some previous studies have described the importance of viral co‐infection on clinical outcome.^57^, ^58^ Bacterial co‐infection among influenza ARIs is also known to be the risk factor for developing severe clinical manifestation.^59^ However, we did not conduct bacteria detection in the current study. Nevertheless, WBC count did not differ among Groups. In future studies, it would be important to elucidate the biological role of bacterial co‐infection on influenza B infections. Lastly, we did not obtain clinical data from those who did not require hospitalization, which made it difficult to estimate the clinical importance of influenza B as well as viral transmissibility. In the future study, we would collect data from mild ARIs and healthy children in the target community to gain a better understanding of the clinical role of influenza B.

## CONFLICT OF INTEREST

The authors have declared that no competing interests exist.

## AUTHOR CONTRIBUTIONS

KA, DAD and LMY designed the study. MT, HMV and EP performed data collection and management. KY, MNL, TO and HAN designed and performed the sample testing. KY, MNL and LMY analyzed and interpreted the results. KY, MNL, KA, HM, MH and LMY wrote and edited the manuscript. All authors reviewed the manuscript and approved the final version of the manuscript.
