# Supplementary material for: Influenza B associated paediatric acute respiratory infection hospitalization in central vietnam
Source: Influenza Other Respir Viruses. 2019 Feb 28;13(3):248–61. doi: 10.1111/irv.12626 (PMC6468073; doi:10.1111/irv.12626)
Supplement: Supplementary file 1 [file IRV-13-248-s001.docx]

# Supplementary Tables

## Supplementary Table 1. Age-stratified prevalence of influenza A and B among paediatric ARI hospitalizations

|  | 2007  (Feb. - Dec.) | 2008  (Jan. - Dec.) | 2009  (Jan. - Dec.) | 2010  (Jan. - Dec.) | 2011  (Jan. - Dec.) | 2012  (Jan. - Dec.) | 2013 (Jan. - Jun.) |
| --- | --- | --- | --- | --- | --- | --- | --- |
| Paediatric ARI hospitalizations (n=4429) | (n=788) | (n=600) | (n=726) | (n=542) | (n=513) | (n=801) | (n=459) |
| Influenza type A (single) (n=496) | (n=127) | (n=77) | (n=90) | (n=36) | (n=51) | (n=48) | (n=67) |
| (Less than 2 years old) (n=281) | 86 (67.7%) | 37 (48.1%) | 47 (52.2%) | 24 (66.7%) | 26 (51.0%) | 31 (64.6%) | 46 (68.7%) |
| (Less than 5 years old) (n=451) | 122 (96.1%) | 65 (84.4%) | 77 (85.6%) | 32 (88.9%) | 47 (92.2%) | 44 (91.7%) | 63 (94.0%) |
| (Older than 5 years old) (n=45) | 5 (3.9%) | 12 (15.6%) | 13 (14.4%) | 4 (11.1%) | 4 (7.8%) | 4 (8.3%) | 4 (6.0%) |
| Influenza type B (single) (n=129) | (n=2) | (n=11) | (n=11) | (n=32) | (n=9) | (n=54) | (n=10) |
| (Less than 2 years old) (n=66) | 1 (50.0%) | 9 (81.8%) | 5 (45.5%) | 16 (50.0%) | 2 (22.2%) | 34 (63.0%) | 6 (60.0%) |
| (Less than 5 years old) (n=109) | 2 (100%) | 10 (90.9%) | 11 (100%) | 25 (78.1%) | 7 (77.8%) | 46 (85.2%) | 7 (70.0%) |
| (Older than 5 years old) (n=20) | 0 | 1 (9.1%) | 0 | 8 (25.0%) | 2 (22.2%) | 8 (14.8%) | 3 (30.0%) |
| Influenza A and B (co-detection) (n=4) | (n=0) | (n=0) | (n=0) | (n=1) | (n=0) | (n=3) | (n=0) |

## Supplementary Table 2. Demographic and clinical characteristics comparison between influenza type A and B

|  | Paediatric ARI hospitalizations due to Influenza A/B during Feb. 2007-June 2013 (n=629) | | |
| --- | --- | --- | --- |
|  | Influenza A ARI hospitalizations (n=496) | Influenza B ARI hospitalizations (n=129) |  |
|  | Total number (%) / Median (IQR) ^¶^ | Total number (%) / Median (IQR) ^¶^ | *p-*value^#^ |
| Demographic information |  |  |  |
| Male sex (%) | 296 (59.7%) | 65 (50.4%) | 0.057 |
| Median age (in month) | 21.4 (IQR: 13.6 - 33.9) | 22.5 (IQR: 12.8 - 48.3) | 0.433 |
| Age group (%) |  |  |  |
| 0 - 12 month | 117 (23.6%) | 34 (26.4%) | **0.002**** |
| 13 - 24 month | 179 (36.1%) | 38 (29.5%) |  |
| 25 - 36 month | 96 (19.4%) | 11 (8.5%) |  |
| 37 - 48 month | 36 (7.3%) | 16 (12.4%) |  |
| 49 - 60 month | 24 (4.8%) | 10 (7.8%) |  |
| > 60 month | 44 (8.9%) | 20 (15.5%) |  |
| Socioeconomic status |  |  |  |
| Daycare attendance (%) | 271 (54.6%) | 59 (45.7%) | 0.065 |
| Family smoking (%) | 254 (51.3%) | **85 (65.9%)** | **0.011*** |
| History |  |  |  |
| Antibiotic used prior to hospitalization (%) | 203 (40.9%) | 50 (38.8%) | 0.492 |
| Underlying medical condition (%) | 194 (39.1%) | 49 (38.0%) | 0.815 |
| Respiratory virus co-infections (%) | 130 (26.2%) | 26 (20.2%) | 0.157 |
| Clinical information |  |  |  |
| Vital sign(s) |  |  |  |
| Median respiratory rate (per min) | 30.0 (IQR: 30.0 - 36.0) | 32.0 (IQR: 29.0 - 36.0) | 0.756 |
| Median body temperature (C.) | **38.5 (IQR: 37.5 - 39.0)** | 38.0 (IQR: 37.5-38.8) | **0.003**** |
| SpO2 (≦90%) | 38 (7.7%) | 5 (3.9%) | 0.171 |
| Respiratory symptom and sign(s) |  |  |  |
| Wheeze (%) | 213 (42.9%) | 51 (39.5%) | 0.485 |
| Tachypnea (%) | 75 (15.1%) | 26 (20.2%) | 0.166 |
| Breathing difficulty (%) | 51 (10.3%) | 8 (6.2%) | 0.179 |
| Crackle (%) | 26 (5.2%) | **19 (14.7%)** | **<0.001***** |
| LRTI and chest X-ray result |  |  |  |
| LRTI^†^(%) | 97 (19.6%) | 25 (19.4%) | 0.964 |
| Mild LRTI (%) | 80 (16.1%) | 17 (13.2%) | 0.580 |
| Severe LRTI^§^(%) | 17 (3.4%) | 8 (6.2%) | 0.208 |
| Abnormal chest X-ray (%) | **172 (34.7%)** | 20 (15.5%) | **<0.001***** |
| Radiologically-confirmed pneumonia (%) | 93 (18.8%) | 15 (11.6%) | 0.078 |
| Treatment and outcome(s) |  |  |  |
| Median onset to hospitalization (in day) | 1.0 (IQR: 1.0 - 3.0) | 2.0 (IQR: 1.0 - 3.0) | 0.169 |
| Median hospitalization duration (in day) | **5.0 (IQR: 3.0 - 7.0)** | 4.0 (IQR: 2.0 - 6.0) | **0.019*** |
| Antibiotic used (%) | 492 (99.2%) | 128 (99.2%) | 1.000 |
| Steroid use (%) | 183 (36.9%) | 56 (43.4%) | 0.175 |
| Blood WBC count (10^3^ cells / uL) | 10.4 (IQR: 7.4 - 13.9) | 9.3 (IQR: 7.0 - 13.1) | 0.115 |
| ^#^All the statistically significant *p*-values are indicated in bold font. As the index for statistically significant values: * are used for *p-*values < 0.05, ** for *p*-values < 0.01, and *** for *p*-values ≦0.001. | | | |
| ^¶^IQR is an abbreviation for Interquartile Range (1st to 3rd). | | | |
| ^†^LRTI is an abbreviation for "lower respiratory tract infection" and based on the WHO definition of clinical pneumonia^24^. | | | |
| ^§^Severe LRTI was defined as the presence of a danger sign, stridor, or chest-wall indrawing. | | | |

## Supplementary Table 3. Seasonal prevalence of WHO Groups in Victoria and Yamagata lineages between February 2007 and June 2013

|  | Year of paediatric ARI sample collection (February 2007 - June 2013) | | | |
| --- | --- | --- | --- | --- |
| Influenza B lineages (Vitoria / Yamagata) (n=91) | 2007 | 2008 - 2009 | 2010 - 2011 | 2012 - 2013 |
| Victoria lineage (n=72) | (n=1) | (n=6) | (n=28) | (n=37) |
| WHO Group(s) |  |  |  |  |
| Group 1 (n=42) | 1 (100%) | 2 (33.3%) | 2 (7.1%) | 37 (100%) |
| Group 4 (n=4) | 0 | 4 (66.7%) | 0 | 0 |
| Group 5 (n=26) | 0 | 0 | 26 (92.9%) | 0 |
| (Group undetermined) (n=0) | 0 | 0 | 0 | 0 |
| Yamagata lineage (n=19) | (n=0) | (n=7) | (n=0) | (n=12) |
| WHO Group(s) |  |  |  |  |
| Group 2 (n=11) | 0 | 3 (42.8%) | 0 | 8 (66.7%) |
| Group 3 (n=7) | 0 | 4 (57.1%) | 0 | 3 (25.0%) |
| (Group undetermined) (n=1) | 0 | 0 | 0 | 1 (8.3%) |

## Supplementary Table 4. Demographic and clinical characteristics comparison between WHO Groups 2 and 3 in Yamagata lineage

|  | Influenza B Yamagata lineage paediatric ARI cases (n=19) | | |
| --- | --- | --- | --- |
|  | WHO Group 2 (n=11) | WHO Group 3 (n=7) |  |
|  | Total number (%) / Median (IQR)^¶^ | | *p*-value^#^ |
| Demographic information |  |  |  |
| Male sex (%) | 7 (63.6%) | 1 (14.3%) | 0.066 |
| Median age (in month) | 22.9 (IQR: 11.6 - 56.2) | 18.5 (IQR: 7.1 - 22.9) | 0.205 |
| Age group (%) |  |  |  |
| 0 - 12 month | 3 (27.3%) | 3 (42.9%) | 0.516 |
| 13 - 24 month | 3 (27.3%) | 4 (57.1%) |  |
| 25 - 36 month | 2 (18.2%) | 0 |  |
| 37 - 48 month | 0 | 0 |  |
| 49 - 60 month | 2 (18.2%) | 0 |  |
| > 60 month | 1 (9.1%) | 0 |  |
| Socioeconomic status |  |  |  |
| Daycare attendance (%) | 2 (18.2%) | 2 (28.6%) | 1.000 |
| Family smoking (%) | 7 (63.6%) | 4 (57.1%) | 1.000 |
| History |  |  |  |
| Antibiotic used prior to hospitalization (%) | 3 (27.3%) | 3 (42.9%) | 0.299 |
| Underlying medical condition (%) | 2 (18.2%) | 3 (42.9%) | 0.326 |
| Respiratory virus co-infection (%) | 2 (18.2%) | 1 (14.3%) | 1.000 |
| Clinical information |  |  |  |
| Vital sign(s) |  |  |  |
| Median respiratory rate (per min) | 34.0 (IQR: 30.0 - 40.0) | 30.0 (IQR: 30.0 - 38.0) | 0.614 |
| Median body temperature (C.) | 38.0 (IQR: 37.5 - 39.0) | 38.5 (IQR: 38.5 - 38.8) | 0.334 |
| SpO2 (≦90%) | 3 (27.3%) | 0 | 0.245 |
| Respiratory symptom and sign(s) |  |  |  |
| Wheeze (%) | 7 (63.6%) | 3 (42.9%) | 0.630 |
| Tachypnea (%) | 1 (9.1%) | 3 (42.9%) | 0.245 |
| Breathing difficulty (%) | 1 (9.1%) | 0 | 1.000 |
| Crackle (%) | 1 (9.1%) | 2 (28.6%) | 0.528 |
| LRTI and chest X-ray result |  |  |  |
| LRTI^†^(%) | 2 (18.2%) | 0 | 0.497 |
| Mild LRTI (%) | 1 (9.1%) | 0 | 1.000 |
| Severe LRTI^§^(%) | 1 (9.1%) | 0 | 1.000 |
| Abnormal chest X-ray (%) | 3 (27.3%) | 2 (28.6%) | 1.000 |
| Radiologically-confirmed pneumonia (%) | 2 (18.2%) | 1 (14.3%) | 1.000 |
| Treatment and outcome(s) |  |  |  |
| Median onset to hospitalization (in day) | 1.0 (IQR: 1.0 - 2.0) | 2.0 (IQR: 0 - 3.0) | 0.600 |
| Median hospitalization duration (in day) | 4.0 (IQR: 2.0 - 5.0) | 2.0 (IQR: 1.0 - 5.0) | 0.433 |
| Antibiotic used (%) | 10 (90.9%) | 7 (100%) | 1.000 |
| Steroid used (%) | 3 (27.3%) | 4 (57.1%) | 0.332 |
| Blood WBC count (10^3^ cells / uL) | 8.8 (IQR: 5.8 - 12.9) | 8.7 (IQR: 7.9 - 12.8) | 0.964 |
| ^#^All the statistically significant *p*-values are indicated in bold font. As the index for statistically significant values: * are used for *p*-values < 0.05, ** for *p*-values < 0.01, and *** for *p*-values ≦0.001. | | | |
| ^¶^IQR is an abbreviation for Interquartile Range (1st to 3rd). | | | |
| ^†^LRTI is an abbreviation for "lower respiratory tract infection", and based on the WHO definition of clinical pneumonia[^55^](#_ENREF_55). | | | |
| ^§^Severe LRTI was defined as the presence of danger sign, stridor, or chest-wall indrawing. | | | |
